# Whipworm secretions and their roles in host-parasite interactions

**DOI:** 10.1186/s13071-022-05483-5

**Published:** 2022-09-29

**Authors:** Rebecca K. Shears, Richard K. Grencis

**Affiliations:** 1 Centre for Bioscience, Manchester Metropolitan University, Manchester, M1 5DG UK; 2 Department of Life Sciences, Faculty of Science and Engineering, Manchester Metropolitan University, Manchester, M1 5DG UK; 3 Lydia Becker Institute for Immunology and Inflammation, Manchester, M13 9PT UK; 4 Wellcome Trust Centre for Cell Matrix Research, Manchester, M13 9PT UK; 5 Division of Infection, Immunity and Respiratory Medicine, Manchester, M13 9PT UK; 6 School of Biological Sciences, Faculty of Biology, Medicine and Health, Manchester Academic Health Science Centre, University of Manchester, Manchester, M13 9PL UK

**Keywords:** Whipworm, *Trichuris*, Excretory/secretory, Extracellular vesicles, Vaccine candidates, Immunomodulators

## Abstract

Whipworm *(Trichuris)* is a genus of roundworms that causes gastrointestinal infections in humans and animals. Of particular interest are *T. trichiura,* the causative agent of human trichuriasis, a neglected tropical disease that affects 477 million people worldwide, and *T. suis*, the pig whipworm species, responsible for growth stunting and economic losses within the agricultural industry. The naturally occurring mouse whipworm, *T. muris,* has been used for decades as a model for trichuriasis, yielding knowledge on the biology of these parasites and the host response to infection. Ex vivo culture of *T. muris* (and to some extent, *T. suis)* has provided insight into the composition of the excretory/secretory (E/S) products released by worms, which include a myriad of proteins, RNAs, lipids, glycans, metabolites and extracellular vesicles. *T. muris* E/S has formed the basis of the search for whipworm vaccine candidates, while the immunomodulatory potential of *T. suis* and *T. muris* secretions has been investigated with the aim of improving our understanding of how these parasites modulate host immunity, as well as identifying immunomodulatory candidates with therapeutic potential in the context of inflammatory diseases. This article will review the various components found within *Trichuris* E/S, their potential as vaccine candidates and their immunomodulatory properties.

**Graphical Abstract:**

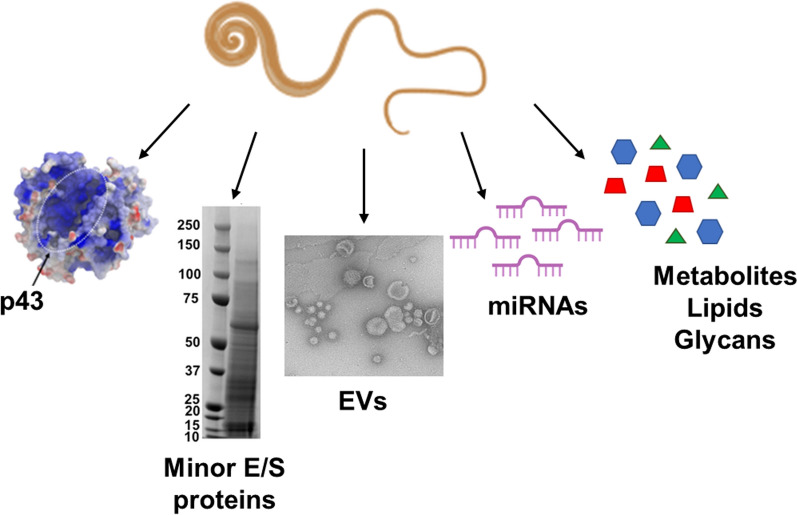

**Supplementary Information:**

The online version contains supplementary material available at 10.1186/s13071-022-05483-5.

## Background

*Trichuris* is a genus of parasitic roundworms that cause infection in humans and other mammals [1]. These worms are also referred to as whipworms due to their whip-like appearance, with a thin end that burrows into the host epithelium and a thicker end that protrudes into the host lumen to facilitate mating and egg release. There are > 70 *Trichuris* species, each of which has its own host species range [1]. The three most well-studied species are *Trichuris muris* (a naturally occurring parasite of mice that has been used for decades as a model of human trichuriasis), *T. trichiura* (the medically important whipworm species that causes trichuriasis in humans) and *T. suis* (pig whipworm, which poses a large economic burden on the agricultural industry) [1–3]. Human trichiuriasis is classed as a neglected tropical disease by the WHO, and *T. trichiura* is one of four soil-transmitted helminths (STHs; named due to their transmission by eggs or hatched larvae that contaminate land in areas where sanitation is poor) that infect humans as well as other closely related primates [3]. *Trichuris* species can also infect companion animals, such as dogs *(T. vulpis*) and cats *(T. serrata* and *T. campanula)* [1]*.*

*T. muris, T. trichiura* and *T. suis* show similarities at the genomic and transcriptomic levels: each species has close to 10,000 genes, and roughly half of these are one-to-one orthologues [4, 5]. Most of our knowledge on the immune response to whipworm infection comes from studying *T. muris* infection in mice (reviewed in [6, 7]), and *T. muris* excretory/secretory products (E/S) have provided insight into potential bioreactive molecules, which may have therapeutic potential as vaccine candidates or immunomodulators in the context of allergic and inflammatory diseases. The study of E/S components also provides insight into the biology of these parasites and how they interact with the host [8, 9].

**Fig. 1:**
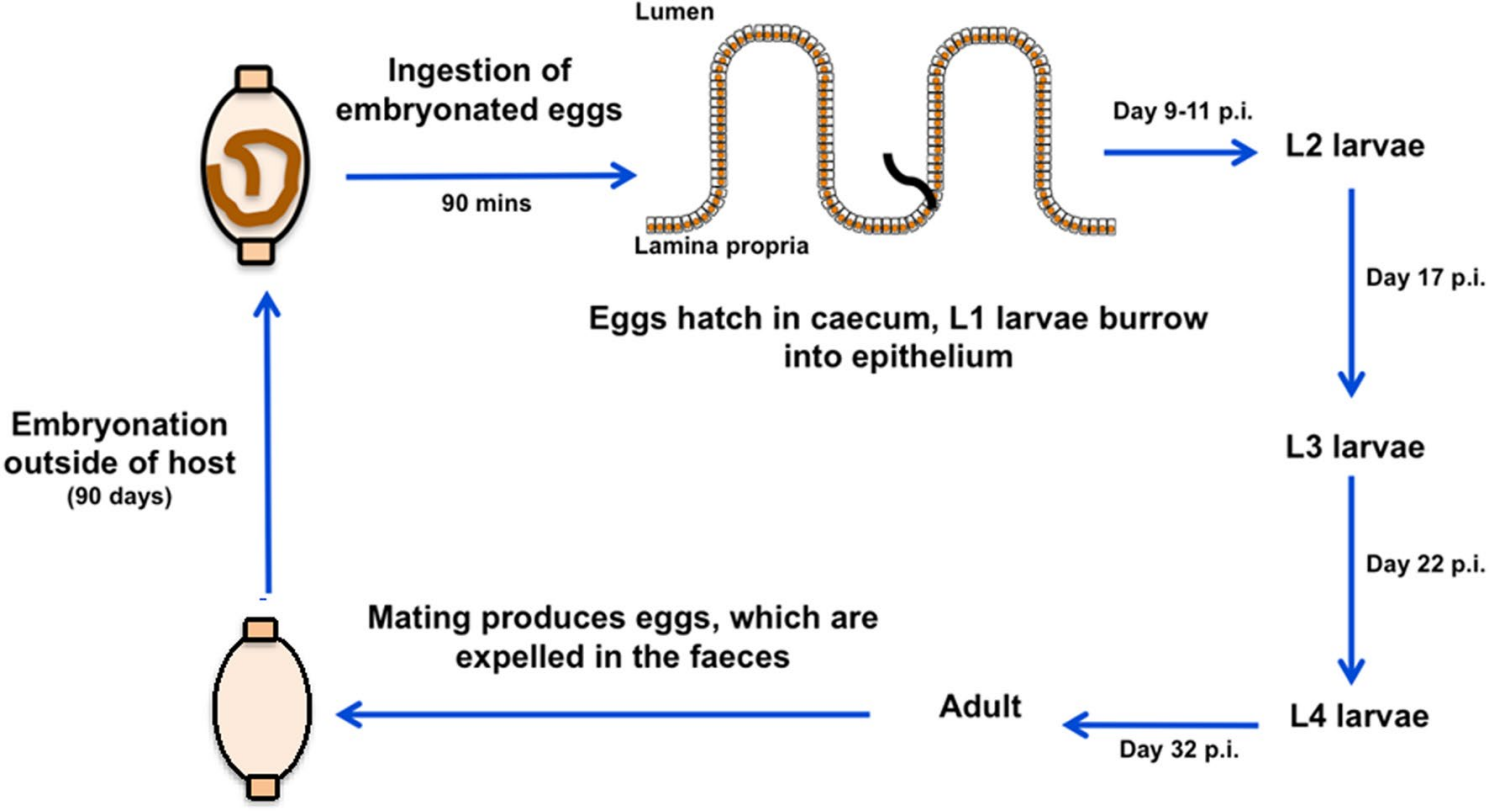
Life-cycle of *T. muris*. Beginning from the top left: infection occurs via the faecal-oral route. Eggs hatch in the host caecum releasing L1 larvae, which then burrow into the caecal epithelial crypts. Larvae undergo four moults to become adults at the time points specified on the diagram. Male and female worms mate and eggs are released into the caecal lumen, where they exit the host in the faeces. Eggs must undergo a period of embryonation before they are infective. L1/L2/L3/L4, First/second/third/fourth-stage larvae, respectively; p.i., post infection

The life-cycle of *T. muris* involves ingestion of embryonated eggs; in the laboratory this can be either a high-dose infection (> 200 eggs, typically leading to acute infection with worm expulsion driven by T helper 2 (Th2) immunity), or a low-dose infection (20–25 eggs, leading to chronic infection and Th1 polarisation of the immune response) [6, 7]. The eggs hatch in the caecum in response to host microbiota components, releasing first-stage (L1) larvae that subsequently burrow into the host epithelium [10]. The L1 larvae undergo four moults to become adults, and the posterior end of the worm starts to protrude out into the lumen from the third-stage (L3) larvae stage onwards [1]. Adult male and female worms mate, and females release eggs into the lumen, which are expelled in the faeces. A period of embryonation outside the host is required before the eggs are infective (Fig. 1). While the life-cycle of other *Trichuris* species follows the same steps, the ease of propagating *T. muris* through laboratory mice, along with the availability of genetically modified mice and a wealth of immunological resources, have contributed to the popularity *T. muris* as a model for whipworm infection [11, 12]. In addition, ex vivo culture of worms has enabled researchers to collect E/S products from adult and larval stages of *T. muris*, enabling the study of various E/S components (particularly the soluble protein content and extracellular vesicles [EVs]) and investigate their immunogenic and immunomodulatory potential [9, 13–15]. *T. suis* E/S products have also been studied in some detail, particularly in the context of identifying immunomodulatory material with possible therapeutic value. In following sections of this review, we provide an overview of the composition of *Trichuris* E/S products and discuss the potential immunogenic and immunomodulatory components within this material.

## Main text

### Whipworm E/S components

Multiple studies, mostly using adult parasites, suggest that *T. muris* E/S contains somewhere between 150 and 500 soluble proteins, as well as EVs, which themselves contain at least 70 proteins and 14 micro-RNAs (miRNAs) [14–16]. The difference in the number of proteins found within the soluble portion of *T.* *muris* E/S is likely due to differences in collection conditions between the studies and the sensitivity of mass spectrometry: proteins present at low abundance are more difficult to detect within complex mixtures of proteins (such as E/S products). However, by fractionating E/S into three different pools using gel filtration chromatography, Shears et al*.* [14] were able to detect > 460 proteins within adult* T. muris* E/S [14]. A proteomic study of two batches of *T. muris* E/S identified some differences between the protein content of different batches, although roughly 60% of proteins were identified in both samples [15]. Most studies on *T. muris* E/S have utilised adult E/S; however, qualitative comparison of the composition of L3 larval and adult *T. muris* E/S found that the proteomic profiles looked similar, with perhaps a few differences in the quantities of the major E/S component, p43 (more abundant in adult E/S), and protein bands at 34, 75, 125 and 170 kDa (more abundant in L3 E/S) [13]. Similarly, there appears to be significant overlap between the proteomic content of *T. suis* second-stage (L2) larvae, L3 larvae, fourth-stage (L4) larvae and adult E/S, with at least 350 proteins identified in total [17].

The single most abundant protein within adult *T. muris* E/S is a 43-kDa poly-cysteine and histidine tailed protein, referred to as p43 [18]. The p43 protein has been shown to bind interleukin (IL)-13, inhibiting the function of this key effector cytokine responsible for *T. muris* expulsion (through de novo production of the intestinal mucin, Muc5ac, and increasing the rate of epithelial cell turnover) [18–20]. p43 can be detected in the caecal mucus of chronically infected mice (the caecum being the primary infection site during chronic infection), suggesting that tethering to mucus proteoglycans may enable p43 to sequester IL-13, thus promoting parasite survival. Another possible role for p43 involves sequestering metal cations using its poly-histidine tail, although this requires further investigation [18].

Gene ontology analysis suggests that trypsin domain-containing proteins are also amongst the most common proteins within the *T. muris* E/S; 21 of the 148 proteins identified by Eichenberger et al*.* [15] and 21 of the 468 proteins identified by Shears et al*.* [14] are trypsin domain-containing proteins [14, 15]. Proteases were also amongst the most common gene ontology term amongst proteins identified in *T. suis* E/S [17]. Eichenberger et al*.* [15] and Shears et al*.* [14] also identified seven to nine cysteine-rich secretory proteins (CAPs) within *T. muris* E/S [14, 15]. These proteins may warrant further study as possible vaccine candidates and/or immunomodulators, given that the historical hookworm vaccine candidates, *Ancylostoma*-secreted protein (ASP) and* Necator americanus* (*Na*) secretory proteins* Na*-ASP-1 and -2 are members of the CAP superfamily. These proteins are speculated to play immunomodulatory roles during the early stages of hookworm infection [21–23]. CAP proteins are also highly abundant in other helminths, including the well-studied rodent hookworms *Heligosomoides polygyrus* and *Nippostrongylus brasiliensis,* which have 25 and 37 CAPs, respectively (referred to as venom allergen-like (VAL) proteins in *H. polygyrus*) [24, 25].

Eichenberger et al*.* [15] noticed that only 40% of proteins identified in *T. muris* E/S contained a signal peptide, which led the authors to investigate whether there were EVs within this content, as the importance of EVs for delivering proteins and miRNAs to host cells has been reported for other helminths, notably *H. polygyrus* [26]. EVs that fit the size and shape characteristics of exosomes have been isolated from *T. muris* E/S by several groups, and from E/S collected from *T. suis* L1 larvae [15, 16, 27, 28]. Between 70 and 364 T*. muris* proteins were identified within *T. muris* EVs. The difference in the number of identified proteins may reflect differences in EV isolation protocols; either way, the vast majority of these (67–88%) lacked a signal peptide, suggesting non-classical secretion within EVs [15, 16, 27]. p43 was amongst the top three most abundant EV proteins identified by both Eichenberger et al*.* [15] and Tritten et al*.* [16] (although it was absent from the study carried out by Shears et al*.* [27]), and a glyceraldehyde-3-phosphate dehydrogenase protein was found within the top 25 most abundant EV proteins in all three studies [15, 16, 27].

Between 14 and 54 miRNAs (as well as 475 full-length messenger RNA [mRNA] transcripts) have been identified within *T. muris* EVs [15, 16, 29]. These parasite-derived miRNAs have been shown to target host mRNAs, including mechanistic target of rapamycin (*mTOR*),* Gata3* (the master regulator of Th2 differentiation) and *D1ertd622e* (encoding macrophage immunometabolism regulator) [30]. Studies in other helminths, most notably *H. polygyrus,* suggest that parasite-derived miRNAs can silence complementary host mRNA by binding to and recruiting Agronaut proteins to these targets, and thus represent a previously undiscovered mechanism by which helminths can regulate the host immune response to promote their own survival. *Heligosomoides polygyrus*-derived miRNAs have been shown to downregulate the expression of *DUSP1,* a key regulator of mitogen-activated protein kinase (MAPK) signalling [26]. *Brugia malayi* (a filarial nematode causing lymphatic filariasis in humans) miRNAs have also been shown to target the mTOR pathway in human dendritic cells [31]. The ability of helminth-derived miRNAs to silence host genes is not restricted to nematodes, with miRNAs derived from *Schistosoma japonicum* EVs able to manipulate host signalling pathways, leading to increased host macrophage proliferation and tumor necrosis factor alpha (TNF-α) production as well as downregulation of nuclear factor kappa B (NF-κB) activity in T cells [32, 33]. Demonstration that *T. muris* EVs can be internalised by caecal and colonic organoids and can alter their transcriptional profile (specifically 88 upregulated and 173 downregulated genes, including genes involved in cytosolic sensing of nucleic acids and type-I interferon signalling) suggest that *Trichuris*-derived EV cargo (both proteins and miRNAs) may play an important role in modulating the host response to promote worm survival in vivo*,* as has been demonstrated for other helminths [34].

In addition to proteins, miRNAs and EVs, metabolomic studies have identified various lipids (short- and long-chain fatty acids [SCFAs and LCFAs, respectively]), amino acids, organic compounds and several sugars and sugar alcohols within *T. muris* E/S [35]. Propionate was found to be the most abundant SCFA within *T. muris* E/S, followed by butyrate and acetate [35]. These SCFAs have been shown to have anti-inflammatory or immunomodulatory activity in multiple models. However, the genome of *T. muris* indicates that the parasite has the capability to produce acetate and formate but not proprionate, butyrate, isobutyrate, valerate, isovalerate or 2-methylbutanoate [4] (Omer Bay, personal communication), suggesting that they may be derived from bacterial species within *T. muris* microbiota (which is known to be distinct from the host microbiota) [9, 36]*.* The dominant saturated fatty acid within *T. muris* E/S is stearic acid which, together with palmitic acid, docosahexaenoic acid, lauric acid and oleic acid, has anti-inflammatory functions, although the functional relevance of these fatty acids within *T. muris* E/S is yet to be fully understood [35]. *Trichuris suis* E/S have also been demonstrated to have antimicrobial properties, and this has been attributed to molecules < 10 kDa in size [37]. Together, these studies suggest that the minor E/S components, particularly lipids and peptides, may provide important insight into the immunomodulatory properties of *T. muris* E/S, and may even hold therapeutic potential.

### The origin of soluble E/S components and EVs

The origin of soluble E/S components and EVs in *Trichuris* species is not yet fully understood. The major *T. muris* E/S protein, p43, appears to be secreted from the longitudinal muscle layer below the cuticle of the parasite [18]; although whether other E/S proteins are secreted from this site remains to be determined. Foth et al*.* [4] identified a subset of genes including serine proteases and protease inhibitors with anterior end-specific expression in *T. muris* and *T. trichiura*, however the precise location from which these proteins are released remains to be determined; it is possible that they are secreted through pores routed in the bacillary band, a structure unique to Trichurid parasites, however they could also enter the digestive tract via the oesophagus and be released into the external environment of the host via the anus [4]. It is interesting to note that the excretory glands of other parasitic and free-living nematodes are located towards the anterior region and release of E/S from the gastrointestinal tract has also been reported [38, 39]. EVs have been postulated to arise from the intestine of the murine gastrointestinal nematode, *H. polygyrus* [26], however the origin of *Trichuris* EVs is yet to be uncovered. Clearly, further research is required to understand the origin of the various components within *Trichuris* E/S—it is if course possible that different components may arise from different structures on or within the worm.

### *Trichuris* E/S as a source of potential vaccine candidates

The potential of E/S as a source of whipworm vaccine candidates has long been recognised, with several research articles dating back to the 1970s, 1980s and 1990s demonstrating the induction of protective immunity against *T. muris* following vaccination of mice via the intraperitoneal, subcutaneous or oral route with native E/S antigens [40–43]. These studies also provided important insights into the suitability of various adjuvants for vaccination against whipworm. Freud’s adjuvant and aluminium hydroxide were both shown to be effective adjuvants for E/S vaccinations administered via the intraperitoneal and subcutaneous route, while co-administration of E/S with cholera toxin showed some efficacy against a high dose infection in BALB/c and C57BL/6 mice, with limited efficacy against infections in B10.BR mice, which are known to be more susceptible to *T. muris* (high-dose infection in this strain results in long-lived chronic infection) [40–43]. More recent studies have shown that vaccination with E/S stimulates a Th2 response in the vaccine and infection-draining lymph nodes [14, 18, 44]. These studies demonstrate that the E/S content of *Trichuris* species is a source of protective antigens; however, little is known about the exact components that are targeted by the immune response during vaccine-mediated immunity.

The most effective single molecule experimental vaccine candidate to date is the major E/S component, p43. Vaccination with native p43 formulated with aluminium hydroxide stimulates protective immunity, with almost complete expulsion of a subsequent low-dose challenge infection [18]. To date, vaccination experiments with recombinant p43 (expressed in baculovirus) are yet to show strong protective immunity against *T. muris* (Bancroft et al*.*, unpublished data)*,* highlighting the challenges of producing recombinant proteins with the ability to induce protective immunity against helminth infections. Other promising immunogenic candidates include a whey acidic protein (*T. muris* whey acidic acid protein [*Tm*-WAP49]), as well as a chymotrypsin-like serine protease and two chitin-binding domain-containing proteins (which were administered as virus-like particles [VLPs] containing T-cell epitopes for these proteins) (Table 1) [45, 46]. Vaccination with *Tm-*WAP49 formulated with Montanide ISA 720 showed efficacy (48% reduction in worm burden) against a high-dose infection in AKR mice, and this was attributed to the induction of antigen-specific Th2 immunity (high levels of IL-5 and IL-13 secretion by splenocytes, and lymphocytes in the vaccine-draining [inguinal] and parasite-draining [mesenteric] lymph nodes) [45]. Vaccination with a highly conserved fragment of WAP49 fused to the hookworm vaccine candidate, *Na-*GST-1 (r*Tm*-WAP-F8 + *Na*-GST-1), resulted in a similarly strong antigen-specific Th2 response from splenocytes, but a weaker antigen-specific Th2 response by inguinal and mesenteric lymphocytes, and a 33% reduction in worm burden [45]. Nevertheless, these recombinant proteins show promise as vaccine candidates, and future studies should seek to evaluate the efficacy of *Tm-*WAP49 in combination with other vaccine candidates, in an attempt boost protective immunity. The study involving r*Tm*-WAP-F8 + *Na*-GST-1 also provides evidence that a pan-helminth vaccine (targeting > 1 STH) may be feasible; however whether this would be possible in practice remains to be determined, especially given the limited efficacy of existing *Trichuris* vaccine candidates and other challenges associated with pre-clinical vaccine trials.

**Table 1:** Pre-clinical *Trichuris* vaccine candidates

Name^a^	UniProt ID^b^	Comments	References
p43	A0A5S6QYG9	Vaccination with native protein induces protective immunity; however, this is yet to be recapitulated with recombinant forms of the protein	Bancroft et al*.* [18]
*Tm-*WAP49	Not listed	Vaccination with recombinant *Tm-*WAP49 induces Th2 immunity, with 48% reduction in worm burden in AKR mice (high-dose infection)	Briggs et al*.* [45]
Chymotrypsin-like serine protease A0A0N5DUC1, chitin-binding domain-containing proteins A0AN5E6C6 and A0A0N5DK22	A0A0N5DUC1, A0AN5E6C6 and A0A0N5DK22, respectively	Vaccination with T-cell epitopes from these proteins incorporated into VLPs resulted in 50% reduction in worm burden (high-dose infection in C57BL/6 mice)	Zawawi et al*.* [46]

*p43* Poly-cysteine and histidine-tailed protein, *Tm*-WAP49 *Trichuris muris* whey acidic acid protein, *VLPs* virus-like particles

^a^Five E/S components have been explored as vaccine candidates using the *T. muris* mouse model (either as single protein vaccines or incorporated into VLPs)

^b^ UniProt identification (ID) is included where available

Zawawi et al*.* [8] employed a novel computational approach to identify potential *Trichuris* vaccine candidates. These authors utilised in silico prediction tools to identify histocompatibility complex class II (MHC-II) molecule T-cell epitopes within the *T. muris* genome, prioritising those with signal peptides (suggesting they may be found within the E/S) for further study [46]. The prioritised candidates included T-cell epitopes found within a chymotrypsin-like serine protease and two chitin-binding domain-containing proteins [46]. All three of these proteins were identified in E/S fractions by Shears et al*.* [14]. The T-cell epitopes were incorporated into hepatitis B core antigen VLPs (an approach that has been explored for other helminths, including *Trichinella spiralis* and *Clonorchis sinensis*) [47, 48] and administered to mice subcutaneously. These pooled VLP vaccinations resulted in a 50% reduction in worm burden at day 14 post infection compared to sham vaccinated mice [46]. These vaccination experiments were performed in C57BL/6 mice, which are naturally resistant to high-dose *T. muris* infections (i.e. worms are expelled before reaching maturity). It would be interesting to investigate the efficacy of these potential vaccine candidates against a subsequent low-dose challenge infection, which is more reflective of a natural infection dose [49].

Shears et al. [27] demonstrated protective immunity (significant reduction in worm burden in low-dose infected C57BL/6 mice) following vaccination of mice with *T. muris* EVs, suggesting that *Trichuris* EVs may represent an alternative source of vaccine candidates [27]. The protection conferred by EV vaccination is dependent on intact vesicles, suggesting that the packaging of immunogenic material within the EV lipid spheres is crucial for their efficacy (notably the authors did not administer the EV vaccine with adjuvant), perhaps as this protects the material from degradation and/or enables slow release of antigen over time [27]. In their study, the authors highlight several potential immunogens (including a vitellogenin-related protein and a TSP-1 domain-containing protein) based on recognition of EV components by host antibodies; however, these will require further study to evaluate their potential as vaccine candidates [27].

### Immunomodulatory potential of *Trichuris* E/S

The immunomodulatory potential of *Trichuris* E/S has gained substantial attention in the last 10 years, partly due to curiosity-driven research to understand how these parasites interact with the host, and partly due to the interest in these parasites (namely *T. suis)* as therapeutic agents against inflammatory bowel disease, multiple sclerosis and other inflammatory disorders (so-called ‘helminth therapy’). Many of the early studies on the immunomodulatory potential of *T. suis* E/S involved investigating the potential of E/S products to modulate allergic or inflammatory diseases in mice. For example, using a murine model of allergic airway disease, Ebner et al*.* [50] demonstrated that administration of *T. suis* L1 larval E/S during the allergen sensitisation phase led to significantly reduced airway hyperreactivity, bronchiolar inflammatory infiltrate and allergen-specific IgE [50]. This effect was shown to be partly dependent on IL-10 induction from a range of immune cells; however, the authors report no significant induction of T regulatory cells, which contrasts with experiments performed with E/S products from other helminths, including *H. polygyrus* E/S (which we now know contains a transforming growth factor beta [TGF-β] homologue) [51]*.*

Kuijk et al*.* [52] demonstrated that administration of *T. suis* E/S to symptomatic mice led to a reduction in clinical disease scores in the context of a murine experimental autoimmune encephalomyelitis (EAE) model (an animal model of multiple sclerosis) [52]. The authors provide mechanistic insight using in vitro assays, demonstrating that *T. suis* E/S products suppress TNF-α and IL-12 secretion by Toll-like receptor (TLR)-activated (with lipopolysaccharide [LPS] or Poly I:C) human dendritic cells (DCs) and supress bacterial-induced IL-17A secretion by memory T cells in vitro [52]*.* A more recent study found that intraperitoneal administration of *T. suis* E/S to rats decreases the severity of EAE and reduces Th17 and Th1 responses in the spinal cord and spleen [53].^.^ *T. suis* E/S has also been demonstrated to alter the phenotype and function of human monocytes by skewing classical monocytes to become anti-inflammatory ‘patrolling’ cells, which exhibit reduced trans-endothelial migration capacity in an in vitro model of the blood–brain barrier [54]. Other studies corroborate these findings, showing that exposure of mouse and human LPS-exposed bone marrow-derived DCs and macrophages to *T. suis* E/S leads to a reduction in pro-inflammatory cytokines, TNF-α and IL-12, and an increase in anti-inflammatory IL-10 [17, 55, 56]. This altered phenotype of *T. suis* E/S-exposed macrophages has been shown to be sustained through epigenetic re-modelling [57].

More recent studies have attempted to identify components within *T. suis* E/S responsible for these modulating effects on macrophages and DCs (summarised in Table 2). Laan et al*.* [55] provide evidence that worm-derived prostaglandin E2 (PGE2) is at least partly responsible for the immunomodulatory properties of *T. suis* E/S. Having previously shown that *T. suis* E/S modulation of DC function is mediated by non-protein components, Laan et al*.* [55] used a fractionation-based approach (column liquid chromatography) to identify potential immunomodulatory components [55, 58]. They narrowed down the search to one fraction within *T. suis* E/S, of which the dominant component was identified as PGE2. The effect of this fraction on the phenotype of LPS-exposed human DCs was similar to that of commercially available PGE2, including decreased expression of the chemokines CCL2, CXCL9, CCL8 and CCL19, increased expression of the chemokine CXCL16 and decreased TNF-α secretion [55]. The authors noted that the highest quantities of PGE2 were found in L4 E/S, although all life-cycle stages produce this lipid compound [55].

**Table 2:** Pre-clinical *Trichuris*-derived immunomodulators that have been tested in vivo or ex vivo

Name	UniProt ID^a^	Comments	References
PGE2	N/A (lipid)	Partial fractionation led to decreased expression of the chemokines CCL2, CXCL9, CCL8 and CCL19, increased expression of the chemokine CXCL16 and decreased TNF-α secretion from human DCs	Laan et al*.* [55]
Triosephosphate isomerase (D918-00,560)	Not listed	Incubation of bone marrow-derived macrophages with recombinant *T. suis* triosephosphate isomerase and nucleoside diphosphate kinase significantly suppressed TNF-α secretion from mouse bone marrow-derived macrophages and DCs	Leroux et al*.* [17]
Nucleoside diphosphate kinase (D918-00,383)	Not listed		
Chitinase KFD48490.1	Not listed	Administration of a recombinant chitinase led to improved clinical symptoms in a murine OVA-induced allergic airway disease model, associated with reduced eosinophil recruitment to the lung	Ebner et al*.* [59]
p43	A0A5S6QYG9	The structural similarity of p43 with part of the IL-13Rα-2 enables it to bind IL-13, a key cytokine for worm expulsion, thus promoting worm survival	Bancroft et al*.* [18]

*DC *Dendritic cell,* IL-13Rα-2* IL-13 receptor subunit alpha-2,* N/A* Not available,* OVA* ovalbumin,* PGE2* prostaglandin E2,* TNF-α *tumor necrosis factor alpha

^a^UniProt identification (ID) is included where available, however in most cases it was not possible to infer this from the information given

Leroux et al*.* [17] identified two worm-derived proteins within *T. suis* E/S with immunomodulatory potential: a triosephosphate isomerase and a nucleoside diphosphate kinase. They too used a fractionation-based approach (size exclusion chromatography) to narrow down the search for immunomodulatory material within *T. suis* E/S that might be responsible for the suppression of macrophage and DC inflammatory responses [17]. These authors showed that incubation of bone marrow-derived macrophages with recombinant *T. suis* triosephosphate isomerase and nucleoside diphosphate kinase significantly suppressed TNF-α secretion from mouse bone marrow-derived macrophages and DCs. They also reported a more prominent effect with adult *T. suis* E/S than with L4 larval E/S, although it is unclear whether this is mediated by differences in the relative abundance of triosephosphate isomerase and nucleoside diphosphate kinase within these secretions, as these data are not included [17]. A standardised concentration of L4 larval and adult E/S were used in these assays. However, there are likely to be differences in the expression profiles and/or relative abundance of bioreactive molecules between different life-cycle stages, and it may be useful to take this into consideration when investigating potential immunomodulators or vaccine candidates within secreted material. Ebner et al. showed that an enzymatically active *T. suis* chitinase identified within L1 larval E/S and with structural similarity to mouse chitinases has immunomodulatory potential [59]. These authors showed that administration of a recombinant version of this chitinase led to improved clinical symptoms in a murine ovalbumin (OVA)-induced allergic airway disease model, associated with reduced eosinophil recruitment to the lung [59]^.^ The authors propose that the mode of action might relate to the structural similarity of this chitinase with host chitinases, although further studies are required to confirm this.

Studies with *T. muris* suggest that the major E/S protein, p43, may also modulate the host response during infection. The structural similarity of p43 with part of the IL-13 receptor subunit alpha-2 (IL-13Rα-2) enables it to bind IL-13, a key cytokine for worm expulsion, thus promoting worm survival [18]. p43 is also capable of tethering to matrix proteoglycans, which may represent a source of bound p43 and thus facilitate sequestration of IL-13 within the gut environment. p43 is able to suppress IL-13-induced RELM-alpha production from peritoneal mouse myeloid cells in vitro and the accumulation of interstitial lung macrophages after intranasal administration of IL-13 in vitro [18]. The p43 protein is expressed by all life-cycle stages of *T. muris*, and homologues of p43 are found in other Trichuridae parasites, including *T. trichiura* and *T. suis*, suggesting that this may be a universal mechanism by which these parasites modulate the host immune response to promote their own survival [18].

*T. suis* E/S has been shown to lead to increased permeability of epithelial monolayers in vitro, suggesting that in vivo secretion of E/S by *Trichuris* species may disrupt the barrier function of the intestinal epithelium, thereby exposing DCs in the lamina propria to E/S products, allowing them to exert their immunomodulatory effects [54]. The authors of this study demonstrate that heat treatment and chymotrypsin digestion do not affect the ability of *T. suis* E/S to disrupt epithelial monolayers in vitro, but that sodium periodate treatment, which oxidises glycan moieties, inhibits the effect of E/S exposure on epithelial integrity, suggesting that disruption of epithelial integrity is mediated by glycans within the E/S [60]. Future studies should seek to identify the specific components involved in this process and the mechanisms by which they disrupt the intestinal epithelium.

Helminth therapy involving *T. suis* infection has been explored in the context of multiple inflammatory disorders, including inflammatory bowel disease, allergic rhinitis and food allergy. Early studies showed potential, but meta analyses have shown no clear benefit of *T. suis* therapy, and it has since fallen out of favour [61, 62]. The ethics of introducing live *T. suis* to humans has also been debated, since the parasite may be able to establish chronic infections, leading to disease. However, harnessing the therapeutic potential of *T. suis* and other helminth derived products may be a more direct way of treating inflammatory disorders, without the risks associated with live infections.

## Conclusions and future directions

There is clear evidence that *Trichuris* secretory products (soluble E/S proteins, lipids and glycans, EVs, RNAs and metabolites) play key roles at the host–pathogen interface (see Additional file 1: Table S1 for summary of the studies describing the protein, miRNA and metabolite content of *T. muris* and *T. suis* E/S and EVs). However, many unanswered questions remain, including the origin of these different components (which may shed light on the biology of these parasites), as well as the identification and evaluation of specific secreted components with therapeutic potential in the context of vaccines and/or treatment of inflammatory disorders.

Various approaches have been used to overcome the challenge of identifying potential vaccine candidates (and immunomodulators) within the hundreds of secreted products, ranging from crude fractionation of E/S to more sophisticated computational processes. However, the limited success of individual vaccine candidates in murine vaccination studies suggests that combining several candidates in a single vaccine might be more efficacious, although there are clear challenges with regards to translating native antigens to effective recombinant vaccine candidates, as highlighted in the context of the major E/S protein, p43, in murine vaccination studies. Purification of native proteins for use in human trials is not feasible for a disease that infects close to half a billion people worldwide due to the requirement for large numbers of susceptible hosts in which to passage the parasite through to collect E/S. Moreover, ex vivo culture of *T. trichiura* and collection of E/S products is challenging as the parasite can only be harvested from humans by administering anthelminthic drugs, which damage/kill the worms, reducing their capacity to secrete E/S. While the use of primate models could be considered in this regard, the amount of native p43 (or other protein of interest) required to vaccinate all those at risk of trichuriasis would still limit the feasibility of this approach. A better understanding of post-translational modification of helminth-secreted proteins is an important step in producing effective recombinant vaccine candidates, or indeed modifying existing candidates to improve their in vivo efficacy.

Exploring the secretome of larval stages further may generate important insights into potential vaccine candidates. Components secreted by multiple life-cycle stages may make better vaccine candidates, especially as human and animal hosts are likely to be infected with multiple life-cycle stages at one time, rather than receive a single bolus infection, as per experimental murine vaccination studies. ‘Trickle’ infection, whereby individuals receive multiple low-dose infections over time, has been modelled in laboratory mice, shedding light on immunity to *Trichuris* infection in situ. Specifically, there is a shift from Th1 to Th2 immunity over time following multiple low-dose infections [63]*.* Moreover, producing a vaccine that induces protective immunity in already-infected individuals is a significant challenge for vaccine design. In the *T. muris* system, the challenge may involve re-directing an ongoing *Trichuris-*specific Th1 response (generated in response to a low-dose infection) towards Th2 immunity, which is required for worm expulsion [43]. Certainly, in this model, it has been shown that animals that have a chronic (low-dose) infection cleared by anthelmintic are still fully susceptible to challenge, even with a high dose of eggs which, if given as a priming infection, would induce solid resistance to challenge (either high- or low-dose infection) [49].

To increase the chance of bringing effective *Trichuris* vaccine candidates to trial, rigorous testing of candidates in the pre-clinical stages is key. Thus far, there has been no standardisation of experimental vaccine protocols, and whilst it is important that researchers trial candidates in different delivery systems (such as VLPs or paired with different adjuvants), it would be helpful to define experimental vaccine protocols to ensure rigorous testing of candidates, namely by standardising infection dose and the length of time between vaccination and infection. Low-dose infection arguably allows more rigorous testing as vaccine candidates need to drive the host immune response towards a Th2 response (rather than a Th1 response which ordinarily prevails following low-dose infection in the absence of vaccination, resulting in chronicity of infection) [49]. Induction of immunological memory is important for an effective vaccine candidate, and a vaccination schedule that leaves at least 3–4 weeks between the primary vaccination and infection would perhaps allow for more rigorous testing of vaccine candidates [64–66]. We also recommend that the UniProt identification is included when publishing data on bioreactive molecules with therapeutic potential. This would enable other researchers to investigate these proteins of interest further, either in combination with other potential vaccine candidates or in other disease models in the case of molecules with immunomodulatory properties. UniProt IDs should remain consistent over time, unlike WormBase accession codes, which have changed with the latest versions of the genome sequences.

Evaluation of *Trichuris*-derived immunomodulators with therapeutic potential against inflammatory disorders has lagged behind that of potential vaccine candidates. Further work should be carried out to evaluate the therapeutic potential of recombinant immunomodulators in a variety of in vivo models, in much the same way that recombinant vaccine candidates are being tested in experimental vaccination studies. However, as some of the immunomodulatory candidates (such as the chitinase and PGE2 identified in *T. suis* E/S) are likely to have structural similarity with host components (allowing them to exploit the same immunomodulatory pathways), care should be taken to avoid unwanted autoimmune reactions and other adverse effects [55, 59]. The *H. polygyrus*-derived TGF-β mimic, referred to as TGM, has shown great promise as an immunomodulator in the context of inflammatory disorders [51]. TGM is structurally unrelated to TGF-β but is capable of binding to the mammalian TGF-β receptor, inducing the expression of Foxp3, the transcription factor typically associated with anti-inflammatory regulatory T cells. Since TGM shares no homology with mammalian TGF-β family members, there should be fewer issues concerning autoimmunity, although there may still be concerns regarding the specificity of the induced anti-inflammatory response and the implications for concomitant infections, which will need to be addressed during preclinical testing [51]. The goal would be to identify similar helminth-derived immunomodulators in other species, which could be of therapeutic value in the context of inflammatory disease. In addition, more research into the role of EV-derived miRNAs in the context of host-pathogen interactions could yield novel therapeutics and this exciting area of research should be explored further.

## Supplementary Information

**Additional file 1: Table S1.** Summary of papers describing the protein, RNA and metabolite profiles of *T. muris *and *T. suis *E/S and EVs. The reference for each study is included for ease of locating these data sets.

## Abbreviations

ASP: *Ancylostoma*-secreted protein
EAE: Experimental autoimmune encephalomyelitis
E/S: Excretory/secretory products
EV: Extracellular vesicle
CAP: Cysteine-rich secretory protein
miRNA: MicroRNA
*Na*: *Necator americanus*
p43: 43-kDa poly-cysteine and histidine-tailed protein
PGE2: Prostaglandin E2
SCFA: Short-chain fatty acid
STHs: Soil-transmitted helminths
*Tm*-WAP49: *Trichuris muris* whey acidic acid protein (49 kDa)
VAL: Venom allergen like
VLPs: Virus-like particles
